# Stafne’s bone defect in a metastatic prostate cancer patient: A diagnostic conundrum

**DOI:** 10.4317/jced.53656

**Published:** 2018-01-01

**Authors:** Wagner-Gomes da Silva, Aristilia-Tahara Kemp, Alan-Roger dos Santos-Silva, Maria del Pilar-Estevez Diz, Thais-Bianca Brandão

**Affiliations:** 1 DDS, MSC, Dental Service, Instituto do Câncer do Estado de São Paulo, Sao Paulo, Brazil; 2 DDS, PhD, Oral Diagnosis, Piracicaba Dental School, University of Campinas, Piracicaba, São Paulo, Brazil; 3 MD, PhD, Clinical Oncology Department, Instituto do Câncer do Estado de São Paulo, São Paulo, Brazil

## Abstract

Stafne’s bone defect (SBD) is an uncommon bone alteration that affects the mandible and usually presents as an asymptomatic radiolucency located in the posterior region of body or angle of the mandible, below the alveolar canal. Although clinical and radiographic features are more often sufficient for the diagnosis, other lesions and bone alterations have been described in the differential diagnosis and may lead to a misinterpretation and an incorrect diagnosis. Herein, we report a case of an 89-yearold man with metastatic prostate cancer to multiple bones, presenting an asymptomatic solitary well-defined radiolucent image on the right side of the posterior body of the mandible, in close contact with its inferior border. A bone depression was confirmed by computed tomography scans of the mandible and a metastatic inclusion was ruled out by bone scintigraphy with a final diagnosis of SBD. The aim of this report was to highlight the importance of differentiating SBD from metastases in cancer patients and to reinforce the usefulness of multiple imaging modalities in the differential diagnosis of SBD.

** Key words:**Stafne’s bone defect, Mandible, Depression, Metastases, Imaging modalities.

## Introduction

Stafnes’s bone defect (SBD) also known as Stafne’s bone cyst/cavity, salivary gland lingual mandibular bone depression and lingual cortical mandibular bone depression, among others names, is an asymptomatic condition affecting the mandible that was first described by Stafne in 1942 (1). The etiology of this bone defect remains controversial, with some authors that believe in congenital or embryonic causes (1,2) and other groups that accept a bone resorption process caused by pressure from submandibular or other glandular tissues (3).

SBDs are usually well-defined unilocular radiolucencies comprising the posterior part of body or angle of mandible (3), although anterior cases have been also reported (4). Panoramic radiographs in addition to clinical features are usually sufficient for the diagnosis of classical cases; however, computed tomography (CT) can be helpful to demonstrate the bone lingual depression (5,6). Less commonly, sialography (7), as well as surgical exploration (3), can be used to confirm the presence of salivary gland tissue in regard to characterize SBD. These procedures aim to exclude other potential lesions in the differential diagnosis, especially, in atypical cases and anterior lingual mandibular bone depressions (8).

Herein, we report one case of a patient with primary prostate adenocarcinoma with multiple bone metastases, who presented an asymptomatic unilocular radiolucent cavity in the right posterior body of the mandible diagnosed as a SBD.

## Case Report

An 89-year-old male patient was referred to oral examination after starting intravenous (IV) bisphosphonate therapy for the treatment of bone metastatic prostate adenocarcinoma. The patient was diagnosed with a prostate adenocarcinoma Gleason 7 (4+3) confirmed by biopsy and presented a PSA level of 14.25 μg/L, complaining of urinary obstructive and irritative symptoms. After diagnosis, the patient was submitted to a transurethral prostatectomy and radical orchiectomy in April of 2012. Initial treatment with bicalutamide (50mg/2xdaily) and tamsulosin (0.4mg/daily) was started after castration and further exchanged by palliative docetaxel (75mg/m2) chemotherapy associated with prednisone (10mg/daily) due to disease progression. Additional drugs included cholecalciferol, calcium carbonate and IV pamidronate (60mg/monthly). The medical background did not include any known previous comorbidity.

**Figure 1:**
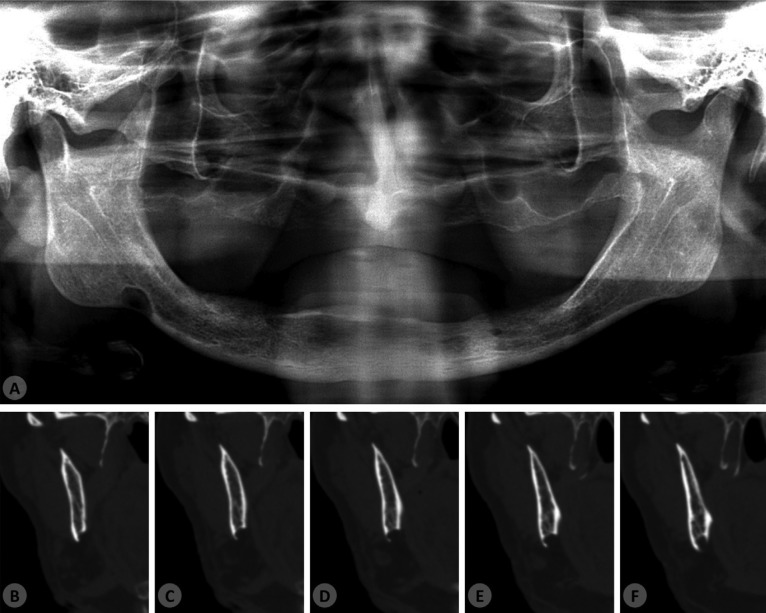
(A) Panoramic radiography showing a well-defined unilocular radiolucent area in the right posterior portion of the mandible near to the angle and in close contact to the inferior border. (B-F) Coronal CT views demonstrating the bone concavity affecting the lingual and inferior aspects of the mandible.

**Figure 2:**
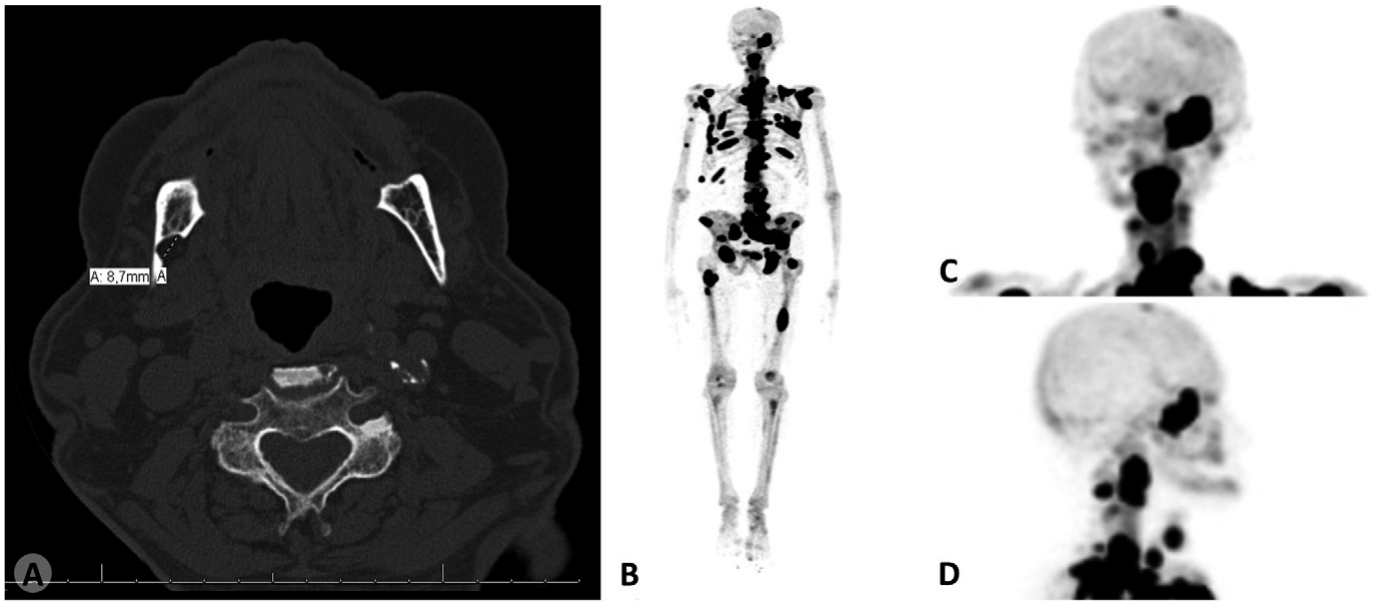
(A) Axial CT view showing the lingual bone depression. (B) Multiple bone metastases evidenced by F-18 radionuclide (dark areas) in a total body bone scintigraphy. (C-D) Detail, showing the lack of hyperconcentration in the correspondent right posterior mandibular area of frontal and lateral cranium views.

Neither facial asymmetry, nor intraoral swelling was observed in the clinical examination; nevertheless, a slight depression could be detected touching the angle of the mandible. The panoramic radiography of the patient revealed a well-defined unilocular image with sclerotic borders at the posterior body of the mandible, below the mandibular canal (Fig. 1A). The patient had no complaints referring to the mandible and denied local trauma or jaw surgery in his medical history. There was no pain or other related symptoms, such as a history of bone expansion in the respective area. To confirm the presumed diagnosis of SBD and help to exclude a possible metastatic lesion to the mandible, a bone window CT scan was requested (Fig. 1B-F). Sections of the exam revealed the presence of a round-shaped, hypodense and well-delimited concavity at the lingual aspect of the mandibular cortical bone with 8,7mm in its greater diameter (Fig. 2A).

Total body skeletal radionuclide imaging with [18F] sodium fluoride (F-18), which was conducted for bone disease screening, revealed numerous osseous metastases in the skull base portion of the sphenoid bone, all segments of vertebral spine, sternum, bilateral ribs, scapulae, right humerus, pelvis and bilateral femurs, including the left femoral neck (Fig. 2B). However, no hyperconcentration of the radiopharmaceutical was evidenced in the jawbones including the mandibular defect (Fig. 2C-D). These features led to the final diagnosis of SBD.

The patient was kept in careful follow-up and the SBD remained unchanged and asymptomatic for six months after the diagnosis. The patient died as a result of cancer progression 19 months after the diagnosis of metastatic bone disease.

## Discussion

SBDs are rare asymptomatic bone defects which mainly affect the lingual mandibular cortical bone below the mandibular nerve and are usually diagnosed in routine panoramic radiographs (3). The patient of the present case was referred to oral examination after the initiation of bisphosphonates therapy but lack any oral complaint. Although a slight depression could be accessed on the head and neck physical examination of the correspondent mandibular area, this signal is commonly absent and is believed to be restricted to the lesions involving the inferior border of the mandible (9).

A variable prevalence of SBDs has been reported in general population and can range from 0.07 to 0.48% in radiographic studies, configuring an uncommon condition (6). More frequently, adult male patients in the 5th and 6th decades of life are affected by SBDs, similarly to the current case (6). Location can vary from the body and angle until first molars (3) to the region before premolars (4). Extremely rare cases may affect lingual or vestibular ascending mandibular ramus nearby parotid gland (3). Additionally, although unilateral SDBs, as our case, are the most frequent, about 25% of these defects can be bilateral (1,3).

Since its first description by Stafne in 1942, wherein 35 cases were reported (1), different causes have been related to this bone alteration and its etiology remains a controversy. Initially, embryonic and congenital remnant tissues, as salivary glands and cartilaginous tissue were suggested to be entrapped during development and ossification of the mandible which could generate the defects (1,2). Occasional cystic lesions completely surrounded by cortical bone (10), including the lingual aspect, were reported and supported this hypothesis; however, in contrast to this observation, most of the cases perform bone concavities that lack complete enclosure (3). Some authors have also reported well-documented cases in which previous panoramical exams did not show evidence of SBD in patients that were after years diagnosed with the bone defect in a detectable stage (3). In addition, the fact that these cases are more frequently diagnosed in adult patients rather than in youngster undermines the idea of an embryologic malformation (11).

In another hand, many other authors have supported that salivary glands can be responsible for progressive and slow cortical bone resorption caused by pressure, which is reliable since either posterior mandible depressions and anterior cases could be a result of submandibular and sublingual glands respectively (3,12). Some authors believe that there is a compensatory hypertrophy related to a lymphocytic infiltration and reduced secretory efficiency, which increases with age (3), or as part of the general somatic growth of salivary glands (13).

Potential misdiagnoses can occur in cases of SBD and many lesions have been reported in the differential diagnosis. Radicular and residual inflammatory odontogenic cysts, non-inflammatory odontogenic cysts, as odontogenic keratocyst and lateral periodontal cyst, odontogenic tumors, benign salivary gland and neural tumors, simple bone cysts, vascular intraosseous lesions, benign fibro-osseous lesions, central giant cell lesions, hyperparathyroidism, solitary plasmocytomas or “punched-out” multiple myeloma lesions, as well as bone metastases, resulting in the inclusion of this last one in our hypothesis, have been suggested in the literature (8,14).

In comprehensive studies, prostate cancer is the second most common metastatic tumor of jaw bones, after lung cancer, affecting men (15). Maxillary and mandibular metastases usually present as ill-defined lytic lesions, but at least some few cases can consist of well-defined radiolucencies. Prostate and other metastatic malignant tumors to the jaw more often show swelling and facial asymmetry with associated symptoms, such as pain, paresthesia, and bone destruction, however, some cases lacking these features have been also reported making difficult to differentiate it from benign conditions or bone alterations (15). In the current case, no hypercaptation of F-18 was observed in the bone scintigraphy which helped to exclude the possibility of a metastatic tumor to the mandible.

No treatment is regularly needed or indicated to SBDs, but surgical exploration can be used to the symptomatic and atypical cases (4). When biopsy followed by histopathological analysis is performed, normal salivary gland tissue (1) is observed or, less commonly, skeletal striated muscle and neural, vascular, adipocytic and lymphoid tissue (3). The current multimodal analysis rendered a final diagnosis of SBD, avoiding any invasive diagnostic procedure.

In summary, our report highlights the importance of clinicians in examining and diagnosing jawbone lesions and alterations in cancer patients. Proper knowledge of SBD may avoid unnecessary treatment, surgical management, or misinterpretation that would negatively influence the prognosis of late stage cancer patients with diffuse bone metastases, such as the current patient. Finally, the correlation between imaging modalities can be useful to rule out another differential diagnosis of SBD.
